# Interactions of Isophorone Derivatives with DNA: Spectroscopic Studies

**DOI:** 10.1371/journal.pone.0129817

**Published:** 2015-06-12

**Authors:** Marco Deiana, Katarzyna Matczyszyn, Julien Massin, Joanna Olesiak-Banska, Chantal Andraud, Marek Samoc

**Affiliations:** 1 Advanced Materials Engineering and Modelling Group, Faculty of Chemistry, Wroclaw University of Technology, Wyb. Wyspianskiego 27, 50–370, Wroclaw, Poland; 2 Laboratoire de Chimie, CNRS UMR 5182, Ecole Normale Supérieure de Lyon, Université Lyon 1, Lyon, France; University of Quebect at Trois-Rivieres, CANADA

## Abstract

Interactions of three new isophorone derivatives, Isoa Isob and Isoc with salmon testes DNA have been investigated using UV-Vis, fluorescence and circular dichroism spectroscopic methods. All the studied compounds interact with DNA through intercalative binding mode. The stoichiometry of the isophorone/DNA adducts was found to be 1:1. The fluorescence quenching data revealed a binding interaction with the base pairs of DNA. The CD data indicate that all the investigated isophorones induce DNA modifications.

## Introduction

A number of studies have indicated that deoxyribonucleic acid (DNA) can be an interesting material not only for biological aspects but also for applications in photonics and electronics [1], [2]. With this in mind, our group has investigated the nonlinear optical properties of well-known intercalators and minor groove binders, such as ethidium bromide and Hoechst 33258 by Z-scan and two-photon fluorescence light microscopy techniques [3–7] gaining expertise in the field of DNA studies. The binding mechanism of small molecules to biomolecules such as *ds*-DNA, *ss*-DNA and proteins has attracted the attention of many research groups and is an active area in the field of biochemistry and medicinal chemistry [8–12]. DNA, being a biodegradable material can have advantages over synthetic polymers which usually are characterized by a very long degradation time [13]. DNA can act as a host for luminescent chromophores: as an example, the DNA-CTMA complex (CTMA:cetyltrimethylammonium chloride) has been shown to be a good matrix for photonic applications [1]. A DNA chain presents sites suitable for various modes of interaction with small molecules, such as intercalation, groove and external binding [14], [15]. Intercalating agents, containing planar heterocyclic groups which stack between adjacent DNA base pairs, can inhibit DNA replication in rapidly growing cancer cells [16], [17]. The complex formed by intercalation is thought to be stabilized, among other factors, by π-π stacking interactions between the drug (the intercalator) and DNA bases [15]. Intercalators introduce strong structural perturbations to DNA. On the other hand, groove binding molecules complement the shape of the groove via van der Waals interactions [18], [19]. The third mentioned type of interaction, the external binding, refers to electrostatic association between molecules that are charged positively and the DNA phosphate sugar backbone, e.g. cations as Mg^2+^ and Ru(II) complexes that are positively charged, interact electrostatically with the DNA phosphate that is negatively charged [20], [21].

In this context, Massin et al. [22] have synthesized some isophorone derivatives and compared their photoluminescence when embedded in DNA-CTMA and poly(methyl methacrylate) (PMMA) matrices [1]. Although no conclusions on intercalation or groove binding of chromophores in the case of DNA-CTMA matrices could be obtained, they showed that the photoluminescence spectra were related to the different interactions which are established between isophorone derivatives and either DNA-CTMA or PMMA, concluding that those with PMMA were stronger than those with DNA-CTMA. Such interactions influence the luminescence efficiency of the chromophore, therefore it is important to determine their nature. Such knowledge is essential for design of new isophorones that would be able to bind to DNA in an optimal way and would enable design of a new fully biodegradable material for photonics.

The aim of this study was to determine the binding mechanism of three push-pull dipolar chromophores made based on the dicyanoisophorone electron acceptor group and varying by the substituent on the donor side [22]. The studies were performed with salmon testes DNA by using UV-Vis, fluorescence and circular dichroism spectroscopies, under physiological pH conditions (7.25). The UV-Vis and fluorescence data allow one to calculate the apparent binding constant and the coordination mode while the circular dichroism can give important information about the occurrence of DNA conformational changes.

## Materials and Methods

### Synthesis

The synthesis of Isoa, b and c has been described previously [22].

### Apparatus

UV-Vis absorption spectra were recorded on a Cary 60 UV-Vis spectrometer (Agilent Technologies). All measurements were carried out with a 1.0 cm path length quartz cell. Fluorescence analyses were carried out with a Hitachi F-4500 spectrofluorometer equipped with a xenon lamp and a thermostated bath. Circular dichroism spectra were recorded with a Jasco J-815 spectropolarimeter (Jasco Inc, USA) equipped with a Jasco Peltier-type temperature controller (CDF-426S/15). A Metrohm 902 Titrando digital pH meter equipped with Tiamo 2.3 software was used to detect the pH values of the solutions.

### Reagents and preparation of stock solutions

Common reagent-grade chemicals were used without further purification. The stock solution of deoxyribonucleic acid sodium salt from salmon testes, purchased from Sigma Aldrich Chem. Co., was prepared by dissolving an appropriate amount of solid DNA powder in 1 mM sodium cacodylate buffer (pH 7.25). The stock solution was stored at 4°C for 24 hours with occasional stirring and was used after no more than 3 days. The appropriate DNA solution concentrations were determined by absorption spectrometry using the molar absorptivity ɛ_260_ = 13200 M^-1^ cm^-1^. The purity of the DNA was checked by monitoring the ratio of the absorbance at 260 and 280 nm and at 260 and 230 nm giving values higher than 1.8 and 2.2, respectively, thus showing DNA being sufficiently free from protein impurities. Stock solutions were prepared by dissolving appropriate amounts of each isophorone in DMSO to final concentrations of 2.5, 1.25, 0.625 and 0.312 mM. The stock solutions were held protected from light by wrapping the vials with aluminum foil.

### UV-Vis measurements of DNA complex formation

UV-Vis absorption spectra were recorded by adding Isoa, b and c from the stock solutions dropwise to 30.9 μM DNA solution and by keeping constant the isophorone concentration (7.5 μM) with incremental addition of 30.9 μM DNA solution. The spectroscopic measurements at the different isophorone-DNA ratios were made in triplicate, at room temperature, and recorded after three minutes to permit the equilibrium between the species. Appropriate mixtures of DMSO and cacodylate buffer were used as reference.

### Fluorescence measurements

A fixed concentration of each isophorone was titrated with incremental addition of DNA and the fluorescence measurements were performed keeping the excitation and emission band slit width of 5.0 nm and after allowing a three minutes equilibration for each DNA addition. A fluorescence free quartz cell of 1 cm path length was used.

### Circular dichroism measurements

The CD spectra were recorded at room temperature in the wavelength range of 200–700 nm, at different Iso/DNA ratios and constant DNA concentration. Before use, the optical chamber of the CD spectrometer was deoxygenated with dry nitrogen and was held under nitrogen atmosphere during the measurements. Each spectrum was averaged from five successive accumulations.

## Results and Discussion

### Spectrophotometric studies

**Fig 1:**
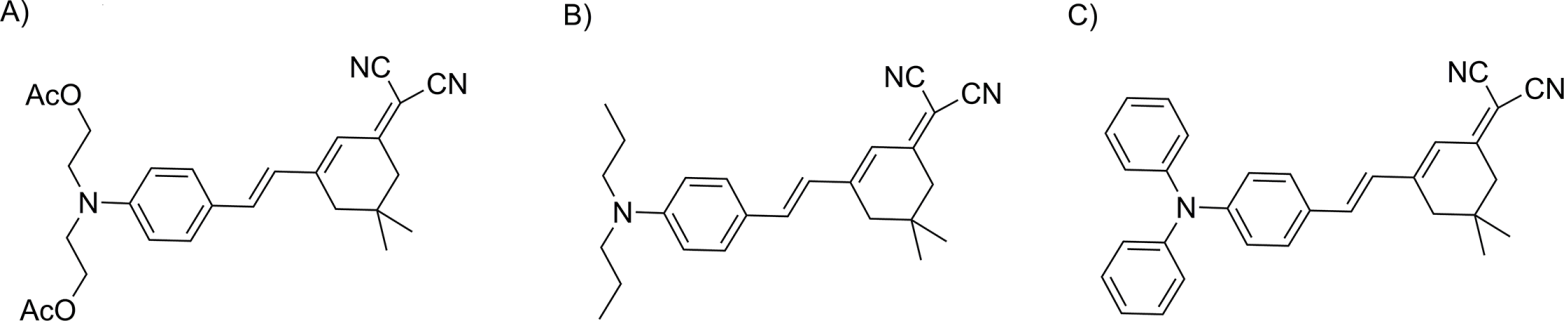
Chemical structures. Structures of: A) Isoa, B) Isob and C) Isoc.

It has been established that the strength of binding of organic molecules (drugs) with DNA helix can be quantified through spectral titration [21], [23–27]. The UV-Vis spectra of the isophorone derivatives, whose structures are reported in Fig 1, are characterized by a strong absorption band in the visible region between 460–560 nm. The UV-Vis spectra of the different Iso-DNA systems show the typical DNA band centered at 260 nm, whose intensity increases as the isophorone concentration increases. Based upon the variation in absorbance at 260 nm, the apparent binding (or association) constant K_a_ of the isophorones with DNA was calculated by using the following equation [15], [28]:

$$1/\left(\text{A}-{\text{A}}_{0}\right)\;=1/\left({\text{A}}_{\infty }-{\text{A}}_{0}\right)+1/\left[\text{Iso}\right]\cdot 1/{\;\text{K}}_{\text{a}}\left({\text{A}}_{\infty }-{\text{A}}_{0}\right)$$ (1)

where A_0_ and A are the absorbances of DNA in the absence and in the presence of the isophorones respectively, and A_∞_ is the final absorbance of the Iso-DNA adduct.

**Fig 2:**
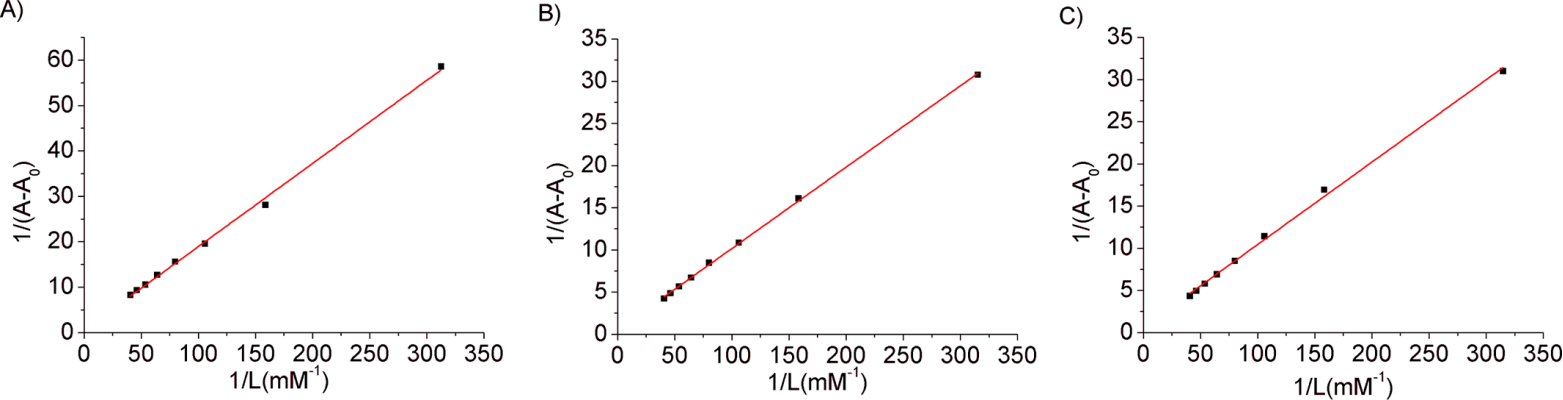
Binding constant determination. Plots of 1/(A-A_0_) *vs*. 1/Isophorone concentration for DNA and their isophorone complexes. A_0_ is the absorbance of DNA (at 260 nm) and A is the absorbance after isophorone addition.

For each isophorone, four systems were studied, keeping constant the concentration of DNA (0.31 mM) treated with different isophorone concentrations. The plots of 1/ (A-A_0_) *vs*. 1/ [Iso] were linear and the binding constant K was calculated from the ratio of the intercept to the slope. As an example, Fig 2 shows the double reciprocal plots of results obtained on adding dropwise the isophorones from the stock solution 0.625 mM to 30.9 μM DNA solution.

The average values of the binding constant for each Iso:DNA complex are reported in Table 1.

**Table 1:** Average values of the binding constants for the studied systems.

Isoa-DNA	Isob-DNA	Isoc-DNA
3.36·10^3^ M^-1^	5.40·10^3^ M^-1^	6.89·10^3^ M^-1^

*Average K values calculated by using the Benesi–Hildebrand equation for all the isophorone-DNA systems studied*.

The K binding constant values reported here are smaller than those typically found for the well-known intercalators such as Methylene Blue-DNA (K = 2.13·10^4^ M^-1^) and Ethidium Bromide-DNA (K = 6.58·10^4^M^-1^) [28], but agree well with those reported for Psoralen-DNA [29], and Naphthoxazole-DNA [30], where intercalative mode of interactions had been assigned.

The active parts of the three isophorones investigated here are exactly the same thus the highest binding constant recorded for Isoc-DNA should be related to its shape as it contains two additional aromatic rings which can increase its planarity providing a greater accessibility inside the base pairs of DNA.

### UV-Vis of the isophorones derivatives-DNA interactions

In order to gain more information on the mode of binding between DNA and isophorones a-c, additional UV-Vis studies were carried out in which the concentration of the isophorones was kept constant (7.5 μM) and the DNA concentration was varied.

UV-Vis spectroscopy can provide important information on the mode of binding of dyes or drugs to DNA. For example, the absorbance peak can shift to longer (bathochromism) or shorter (hypsochromism) wavelengths, indicating structural changes of DNA. Binding to the DNA through intercalation usually results in such behavior. The intercalative mode involves a stacking interaction between an aromatic chromophore and the base pair of DNA and the extent of the hypochromism is related to the strength of intercalative interaction [14], [15]. Hyperchromic (increase of DNA absorbance) and hypsochromic effects can be related to a strong intercalative binding of drugs, such as Methotrexate, and DNA [31]. An intercalative interaction mode has been proposed for Fagaronine, Ethoxidine and Methyl Red, for which a hypsochromic effect was detected after the DNA addition [32].

**Fig 3:**
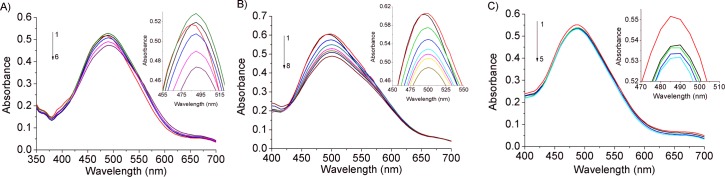
Interaction of the isophorone derivatives in presence of *ds*-DNA. UV-Vis absorption spectra of: A) Isoa (7.5 μM) treated with: 0.0, 1.5, 3.0, 6.0, 7.5, 10.5 μM (curves 1–6) of DNA. B) Isob (7.5 μM) treated with: 0.0, 1.5, 3.0, 4.5, 6.0, 7.5, 9.0 13.5 μM (curves 1–8) of DNA. C) Isoc (7.5 μM) treated with: 0.0, 1.5, 6.0, 7.5, 10.5 μM (curves 1–5) of DNA. All the experiments were carried out at room temperature in sodium cacodylate trihydrate 1 mM (pH 7.25). Inset: Absorption spectra highlights of A) Isoa, B) Isob and C) Isoc with DNA.

Isophorones a and b show an absorption band (λ_max_) at 485 and 495 nm, respectively, which decreases as DNA concentration increases (hypochromic effect) with a red shift of 7 and 5 nm, respectively. On the other hand, isophorone c shows an absorption band (λ_max_) at 490 nm, whose intensity decreases slightly (hypochromic effect) without any band shift (Fig 3). These findings suggest that all the isophorones studied interact with DNA mainly through a stacking interaction between the aromatic chromophore and the base pair of DNA.

### Determination of the binding parameters

**Fig 4:**
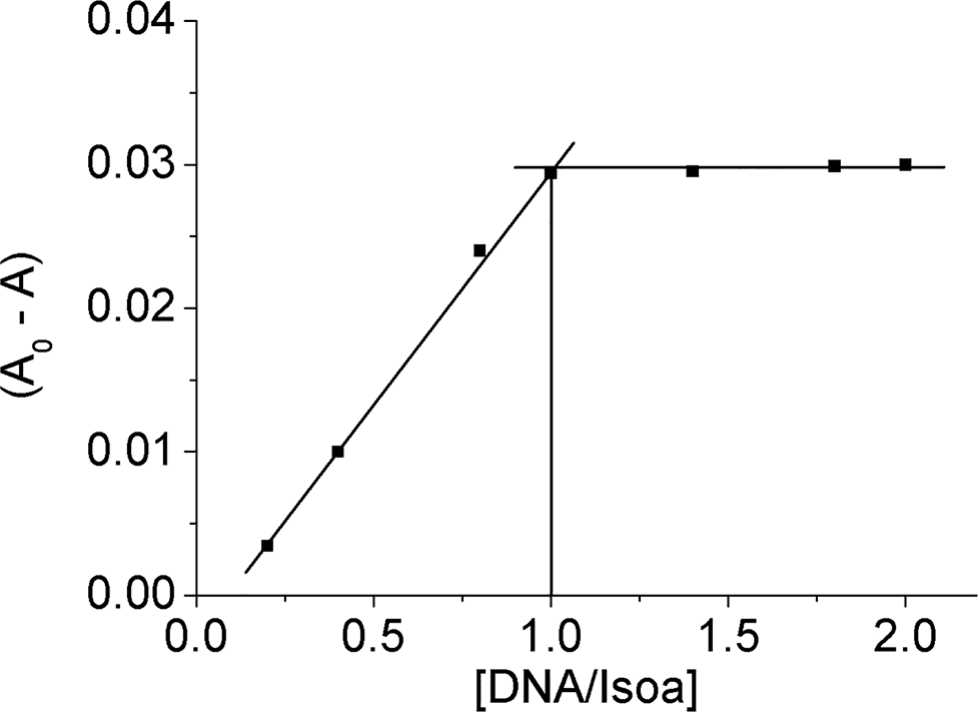
Binding stoichiometry. Mole ratio plot of Isoa-DNA system.

The mole-ratio method was employed to evaluate the isophorone-DNA binding stoichiometries, keeping constant the concentration of the isophorones and varying that of DNA. The plots of the difference in absorption intensity (A_0_-A) at 485 and 495 nm for Isoa and b, respectively, versus the DNA/Iso mole ratio of the corresponding isophorone are indicative of a single binding mode in each case, as revealed by one break point. From the inflection points the molar ratio DNA/Isoa and DNA/Isob results to be equal to 1.0 and 0.9, respectively. Therefore, the number of base pairs of DNA bound per Isoa and b can be estimated to be around 1.0 and 0.9, respectively. This value is in good agreement with that found for the intercalator Naphthoxazole for which a 1:1 stoichiometry was assigned [30]. As an example, Fig 4 shows the mole ratio plot for the DNA-Isoa system. It was not easy to determine the stoichiometry for Isoc due to the slight hypochromic effect of the band at 490 nm. However the plot obtained is in agreement with those found for Isoa and Isob and indicates that the number of base pairs involved in the interaction with Isoc is still one.

### Fluorescence titration studies

**Fig 5:**
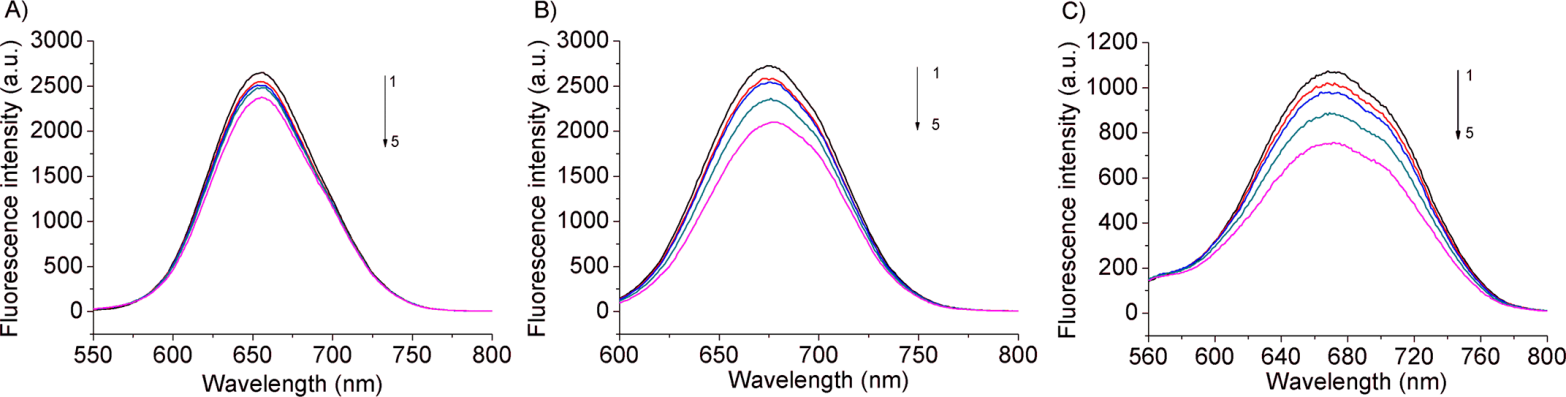
Interaction of the isophorone derivatives with DNA studied using fluorescence spectroscopy. Fluorescence emission spectra of: A) Isoa (15.6 μM) treated with: 0.0, 9.90, 19,80 47.16, 86.21 μM (curves 1–5) of DNA (λ_exc_ = 500 nm). B) Isob (15.6 μM) treated with: 0.0, 9.90, 19.80, 47.16, 86.21 μM (curves 1–5) of DNA (λ_exc_ = 515 nm). C) Isoc (15.6 μM) treated with: 0.0, 9.90, 19.80, 47.16, 86.21 μM (curves 1–5) of DNA (λ_exc_ = 510 nm). All the experiments were carried out at room temperature.

The isophorone-DNA systems were also studied by fluorescence spectroscopy, keeping constant the isophorone concentration and varying that of DNA. The studied molecules show intense fluorescence. The emission spectra of Isoa, b and c (Fig 5) are characterized by a maximum centered at 655, 674 and 672 nm, respectively.

**Fig 6:**
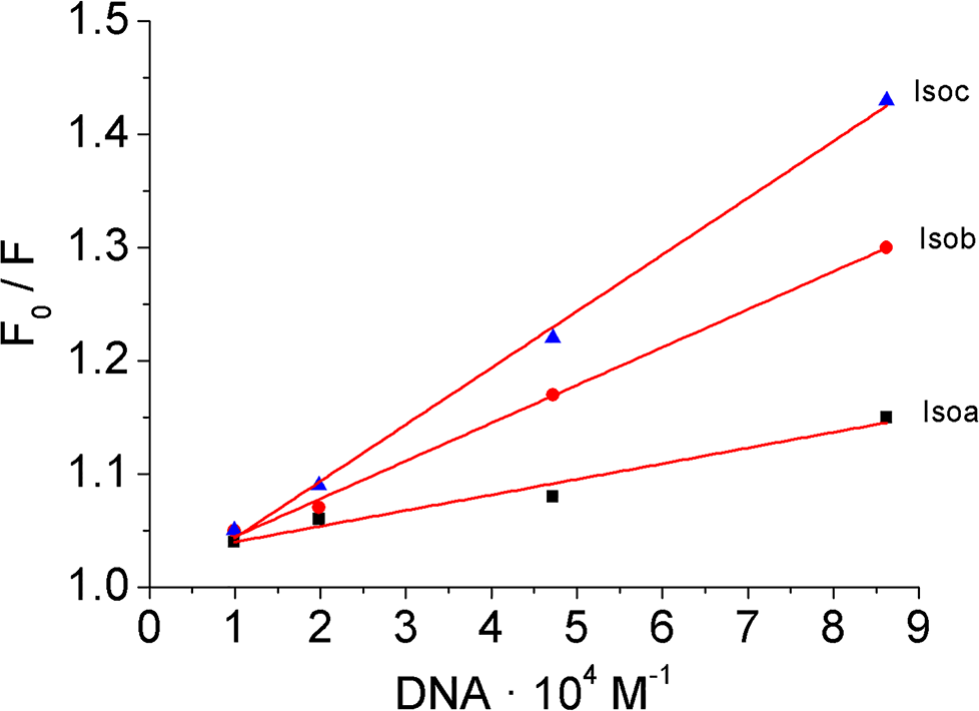
Stern-Volmer plots for the fluorescence quenching of Isoa-c by DNA. Plot of the ratio of the isophorone derivatives fluorescence intensity before and after incremental addition of DNA as a function of the quencher concentration for the determination of the Stern-Volmer quenching rate constant.

With increasing concentration of DNA, progressive quenching was observed for Isoa, b and c revealing a binding interaction taking place. It is well known that the fluorescence quenching can be static, resulting from the formation of a fluorophore-quencher complex or dynamic usually ascribed to the diffusive encounter between the fluorophore and the quencher [33]. In order to distinguish and ascertain quantitatively the possible quenching mechanism the Stern-Volmer equation was used [33], [34]:

$${\text{F}}_{0}/\text{F}=1+{\text{K}}_{\text{q}}\;\tau_{0}\;\left[\text{Q}\right]=1+{\text{K}}_{\text{sv}}\;\left[\text{Q}\right]$$ (2)

where F_0_ and F are the fluorescence intensity in absence and presence of DNA, respectively. K_q_ is the quenching rate constant of the biomolecules, K_sv_ is the Stern-Volmer quenching constant, [Q] is the concentration of DNA and τ_0_ is the average excited-state lifetime of biomolecules without a quencher and it is equal to 10^−8^ s [34]. From the plots of Eq 2 (Fig 6), the values of K_sv_ and K_q_ were obtained for all the Iso-DNA systems studied and are listed in Table 2.

**Table 2:** Stern-Volmer (K_sv_), quenching rate consant (K_q_), association constants (K_f_) and number of binding sites (n) of the interaction between Isoa-c and DNA.

	K_sv_ M^-1^	K_q_ M^-1^ s^-1^	K_f_ M^-1^	n
Isoa	1.38·10^3^	1.38·10^11^	187.6	0.74
Isob	3.35·10^3^	3.35·10^11^	383.7	0.77
Isoc	5.01·10^3^	5.01·10^11^	4507.1	0.99

Determination of the main parameters for the Isophorone-DNA systems calculated by using the Stern-Volmer relationship.

For dynamic quenching, the maximum diffusion collisional quenching rate of various quenchers with biopolymers is about 2.0 × 10^10^ M^-1^ s^-1^ [35]. Since the values of K_q_ were much greater than 2.0 × 10^10^ M^-1^ s^-1^ the quenching can be ascribed to the formation of Iso-DNA complex confirming the static mechanism.

In order to provide further insights in the association process the binding constants (K_f_) and the number of binding sites (n) involved in the Iso-DNA systems were calculated according to the following equation [36]:

$$\text{log}\;\left[\left({\text{F}}_{0}-\text{F}\right)\right]/\text{F}={\text{log K}}_{\text{f}}+\text{n log}\left[\text{Q}\right]$$ (3)

**Fig 7:**
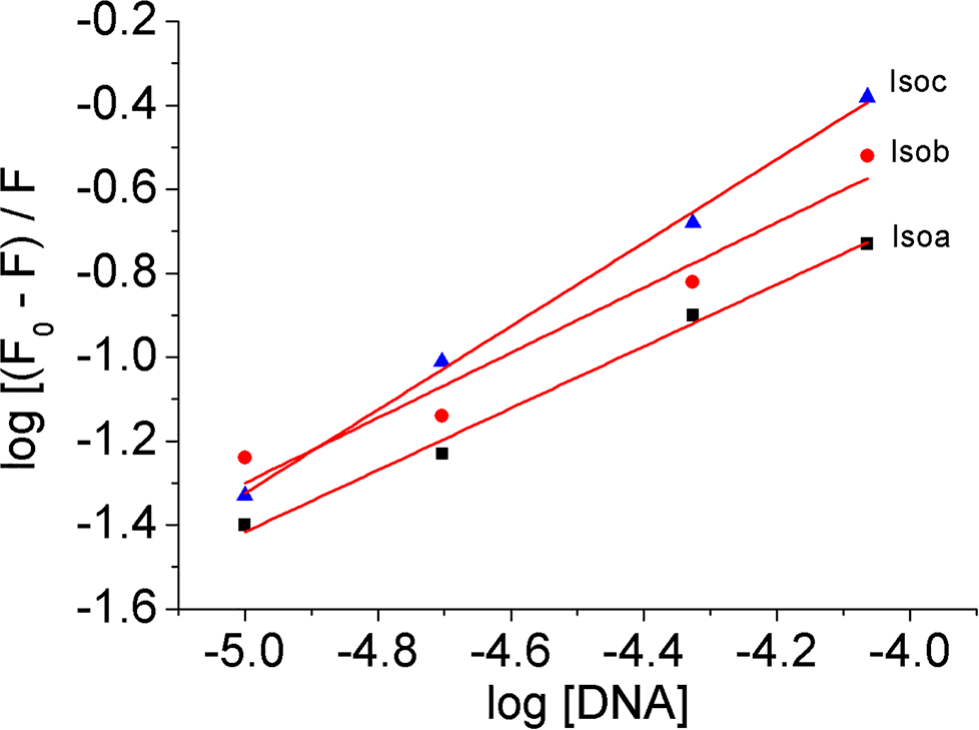
Plot of log [(F_0_-F)/F] *vs*. log [DNA]. Determination of the binding constant and number of binding sites for the Iso-DNA systems.

The plots of log[(F_0_-F)/F] *versus* log[Q] are linear (Fig 7) and the values of K_f_ and n, shown in Table 2 have been obtained from the intercept and the slope, respectively.

The calculated binding constants for the Iso-DNA systems are slightly lower than those calculated by absorption spectroscopy due to the difference in the method used [37]. However, the magnitude of the evaluated association constants still confirms the higher affinity of Isoc, with respect to Isoa and Isob, to the DNA base pairs, which is in agreement with the data derived from UV-Vis spectroscopy.

### Spectroscopic study using circular dichroism

In order to verify if the studied isophorones are able to induce DNA conformational changes, circular dichroism measurements were recorded in the 200–700 nm range with the DNA concentration kept constant and varying that of the isophorones. The CD spectrum of salmon testes DNA is characterized by two bands: a negative one at ≈ 245 nm and a positive one at ≈ 275 nm, which are due to helicity and to stacking interactions among the base pairs [38], and are sensitive to the interaction with isophorones [39]. After addition of the isophorones an ICD band is revealed in each system studied. In particular, bands in the 360–430 nm, 510–525 nm and 470–544 nm range appear in the Isoa, b and c-DNA system, respectively. The induced circular dichroism bands, as found in the investigation of the interaction between the anticancer drug mitoxantrone with DNA, are much smaller in intensity as compared to the positive and negative DNA bands and their variation cannot be determined due to large noise in those regions [40]. However, the presence of these bands reveals that all the isophorone derivatives are able to interact with ds-DNA.

**Fig 8:**
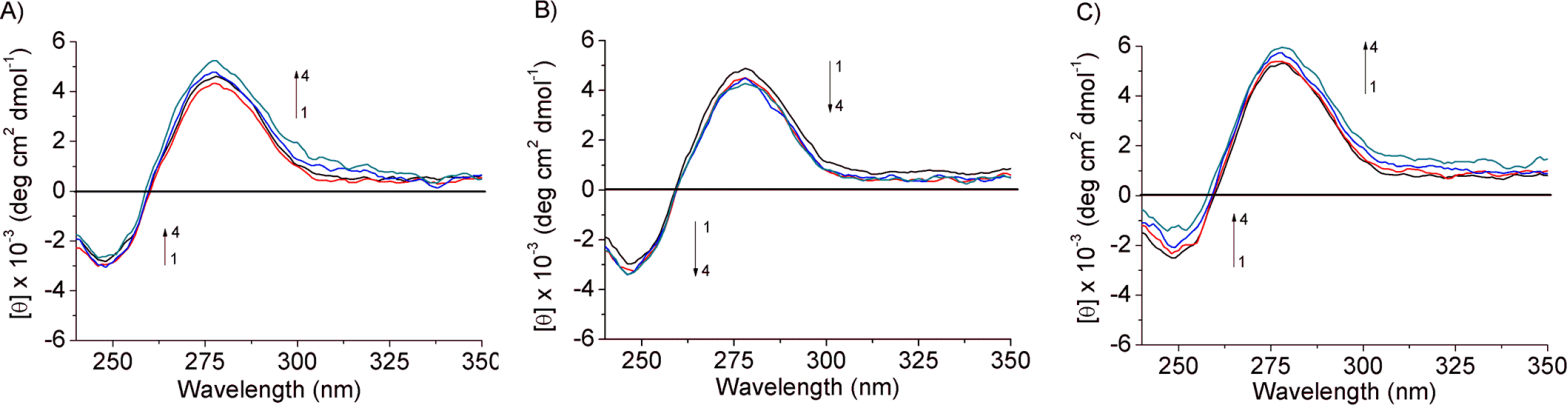
Effect of the isophorone derivatives on the DNA conformation. Circular dichroism (CD) spectra of DNA (30.9 μM) treated with: A) 0.0 (black line), 12.82 (red line), 38.07 (blue line), 62.81(green line) μM (curves 1–4) of Isoa, B) 0.0 (black line), 12.82 (red line), 38.07 (blue line), 62.81(green line) μM (curves 1–4) of Isob, C) 0.0 (black line), 12.82 (red line), 38.07 (blue line), 62.81(green line) μM (curves 1–4) of Isoc.

For Isoa (Fig 8A), the band at 275 nm decreases after the first addition of C_isoa_ = 12.82 μM, but increases linearly up to C_isoa_ = 62.81 μM, which is accompanied by a slight blue shift and a total change in ellipticity of 0.65 mdeg. The intensity of the band at 245 nm decreases linearly up to C_isoa_ concentration of 38.07 μM while an increasing (shifting toward zero) at C_isoa_ 62.81 μM is observed with a total change in ellipticity of 0.20 mdeg. The changes of the CD signal indicate that Isoa interacts with the base pairs of salmon testes DNA through the aromatic rings.

For isoc the bands at 275 and 245 increase continuously (Fig 8C) giving a total change in ellipticity of 0.65 and 1.23 mdeg, respectively, indicating an interaction similar to that recorded for Isoa. The conformational changes induced by both Isoa and c are indicative of an intermediate stage of B-to-A DNA transition in which the B-conformation is still predominant. A similar trend was observed in the study of the interaction between Cyanazine [41] and Psoralen [29] with calf thymus DNA in which intercalation binding had been confirmed.

On the other hand, the bands at 275 and 245 nm decrease linearly after each addition of Isob (Fig 8B) with a simultaneous slight red and blue shift of the positive and negative bands, respectively, indicating the existence of an interaction with nucleic acid.

The results suggest that the destabilization of the B-DNA moiety arises from the insertion of Isob between base pairs of DNA leading to changing the right-handed DNA helicity. Similar conclusions were reported about the interaction of Prodigiosin with ct-DNA [42].

## Conclusions

The data obtained from spectrophotometric measurements indicate that all the studied isophorones interact with DNA, the affinity of Isoc to DNA being the highest. The UV-Vis spectra, recorded by keeping constant the concentration of isophorones and increasing the DNA concentration, suggest that the isophorones interact with DNA mainly through a stacking interaction between the aromatic chromophore and the base pair of DNA with a 1:1 stoichiometry.

The fluorescence data are consistent with the findings drawn from the UV-Vis data and confirm the interactions with DNA.

The CD data clearly indicate that the isophorones induce DNA modifications. In particular, Isoa and c act in a similar way with the base pairs of DNA, on the other hand Isob mainly disturbs the right handed helicity of DNA.

## Abbreviations

Iso: isophorone
DNA: deoxyribonucleic acid
ds: double-stranded
ss: single-stranded
ct: calf thymus
CTMA: cetyltrimethylammonium chloride
PMMA: poly(methyl methacrylate)
CD: circular dichroism
ICD: induced circular dichroism
