# Correction: Interactions of Isophorone Derivatives with DNA: Spectroscopic Studies

**DOI:** 10.1371/journal.pone.0133814

**Published:** 2015-07-20

**Authors:** Marco Deiana, Katarzyna Matczyszyn, Julien Massin, Joanna Olesiak-Banska, Chantal Andraud, Marek Samoc

The following information is missing from the Funding section: Wroclaw Centre of Biotechnology, Programme, The Leading National Research Centre (KNOW) provided funding for the publication of the results of the study.
